# Glucocorticosteroids in Nano-Sterically Stabilized Liposomes Are Efficacious for Elimination of the Acute Symptoms of Experimental Cerebral Malaria

**DOI:** 10.1371/journal.pone.0072722

**Published:** 2013-08-26

**Authors:** Judith H. Waknine-Grinberg, Simcha Even-Chen, Jasmine Avichzer, Keren Turjeman, Annael Bentura-Marciano, Richard K. Haynes, Lola Weiss, Nahum Allon, Haim Ovadia, Jacob Golenser, Yechezkel Barenholz

**Affiliations:** 1 Laboratory of Membrane and Liposome Research, Department of Biochemistry, Institute for Medical Research – Israel-Canada (IMRIC), The Hebrew University - Hadassah Medical School, Jerusalem, Israel; 2 Department of Microbiology and Molecular Genetics, The Kuvin Center for the Study of Infectious and Tropical Diseases, The Hebrew University - Hadassah Medical School, Jerusalem, Israel; 3 Agnes Ginges Center for Human Neurogenetics, Department of Neurology, Hadassah University Hospital, Jerusalem, Israel; 4 Department of Chemistry, Institute of Molecular Technology for Drug Discovery and Synthesis, The Hong Kong University of Science and Technology, Clear Water Bay, Kowloon, Hong Kong; 5 Department of Bone Marrow Transplantation and Cancer Immunotherapy, Hadassah University Hospital, Jerusalem, Israel; Osaka University, Japan

## Abstract

Cerebral malaria is the most severe complication of *Plasmodium falciparum* infection, and a leading cause of death in children under the age of five in malaria-endemic areas. We report high therapeutic efficacy of a novel formulation of liposome-encapsulated water-soluble glucocorticoid prodrugs, and in particular β-methasone hemisuccinate (BMS), for treatment of experimental cerebral malaria (ECM), using the murine *P. berghei* ANKA model. BMS is a novel derivative of the potent steroid β-methasone, and was specially synthesized to enable remote loading into nano-sterically stabilized liposomes (nSSL), to form nSSL-BMS. The novel nano-drug, composed of nSSL remote loaded with BMS, dramatically improves drug efficacy and abolishes the high toxicity seen upon administration of free BMS. nSSL-BMS reduces ECM rates in a dose-dependent manner and creates a survival time-window, enabling administration of an antiplasmodial drug, such as artemisone. Administration of artemisone after treatment with the nSSL-BMS results in complete cure. Treatment with BMS leads to lower levels of cerebral inflammation, demonstrated by changes in cytokines, chemokines, and cell markers, as well as diminished hemorrhage and edema, correlating with reduced clinical score. Administration of the liposomal formulation results in accumulation of BMS in the brains of sick mice but not of healthy mice. This steroidal nano-drug effectively eliminates the adverse effects of the cerebral syndrome even when the treatment is started at late stages of disease, in which disruption of the blood-brain barrier has occurred and mice show clear signs of neurological impairment. Overall, sequential treatment with nSSL-BMS and artemisone may be an efficacious and well-tolerated therapy for prevention of CM, elimination of parasites, and prevention of long-term cognitive damage.

## Introduction

Cerebral malaria (CM) is the most severe pathology caused by *P. falciparum* infection. Approximately 7–11% of all severe malaria cases manifest as CM, typified by fever, impaired consciousness, and signs of neurological damage. The clinical diagnosis of CM requires the presence of coma (Glasgow coma scale <7/15) at least one hour after termination of a seizure or correction of hypoglycemia, detection of *P. falciparum* in blood smears, and exclusion of other potential causes of coma. Disease progression is rapid, with as little as one week between the onset of clinical signs and non-rousable coma. With current treatment options, CM is associated with a mortality rate of 15–30%, and a significant percentage of survivors (10–17%) are left with permanent neurological impairment and cognitive deficits [1].

CM is likely the result of a complex sequence of interrelated events. Current models of human CM postulate a contribution of multiple factors, including microvascular sequestration and blockage leading to local ischemia; cytopathic hypoxia; rupture of parasite-infected red blood cells (iRBC) and the release of parasite-derived toxins; and upregulation of numerous immune or immune-related responses (especially Th1-type responses) - all of which combine to lead to blood-brain-barrier (BBB) breakdown, microglial and astrocyte activation, and subsequent damage or death of microglia, astrocytes, and neurons [2], [3].

Glucocorticosteroids (GC) are the drugs of choice in most diseases with an inflammatory component. However, in many cases their unfavorable pharmacokinetics and biodistribution result in low efficiency and relatively high toxicity [4], which limit their utility. We have recently overcome these major deficiencies by developing steroid-loaded long-circulating nano-liposomal formulations, referred to as steroidal nano-drugs. These are based on the use of water-soluble amphipathic weak acid GC prodrugs, which are remote-loaded at high drug-to-lipid ratios into small (<100 nm) pegylated nano-liposomes, also referred to as nano-sterically stabilized liposomes (nSSL) [5]. These nSSL, like most liposomal formulations, are biocompatible, biodegradable, non-toxic, and lack immunogenicity [6]. In addition, such nano-drugs are unique in their ability to reach inflamed sites in vivo, including the brain [5]. Passive targeting via nSSL takes advantage of the increased permeability of vasculature in inflamed tissues, resulting in improved drug delivery to inflamed areas of the brain (but not to normal, healthy brain) and a reduction in drug side effects related to accumulation at other sites (reviewed in [7]). In addition, nSSL have a long plasma circulation time, a prerequisite for sufficient drug delivery to extra-reticuloendothelial system (RES) disease sites. The presence of a lipopolymer, the pegylated lipid PEG-DSPE, in the liposome membrane reduces interactions with blood components and greatly reduces liposome uptake by the RES [8].

**Figure 1:**
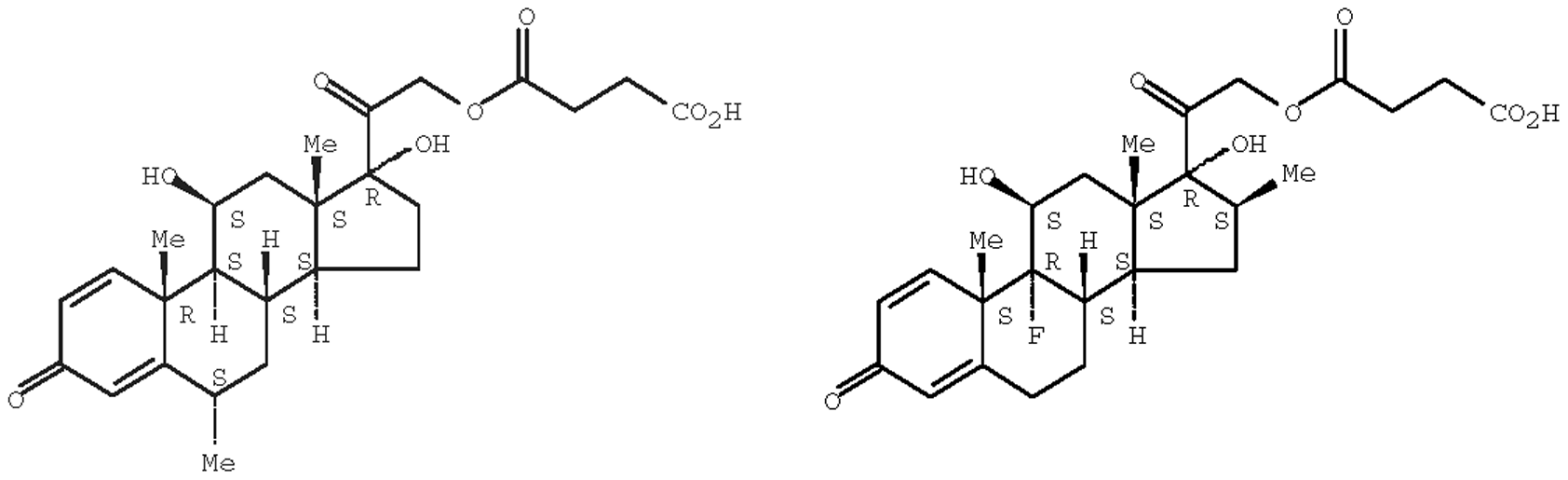
Structures of the glucocorticoid prodrugs methylprednisolone hemisuccinate (MPS, left) and β-methasone hemisuccinate (BMS, right). Both compounds are amphipathic weak acids suitable for loading into nSSL.

Although previous studies have implied a negative impact [9], [10], the topic of using steroids for malaria treatment remains controversial [11], [12]. Utilizing new formulations and treatment schedules, we present results which suggest that the use of steroids may be beneficial for the treatment of cerebral malaria. Based on in silico analysis [13] we selected two GC prodrugs, methylprednisolone hemisuccinate sodium salt (MPS), and β-methasone hemisuccinate (BMS, Figure 1). Both lack mineralocorticoid activity and have high anti-inflammatory potency. MPS, ∼5 times more potent than hydrocortisone, is commercially available and is typically used for its immunosuppressive and anti-inflammatory activities [14]. The second GC produg used, BMS, is not commercially available and was custom synthesized for us. Evaluation of its activity is based on what is known for the parent drug, β-methasone, which is ∼25–30-fold more potent than hydrocortisone [15]. Being water-soluble amphipathic weak acid prodrugs, MPS and BMS can be remote loaded into liposomes by an intraliposome-high/extraliposome medium-low transmembrane calcium acetate gradient. This loading procedure results in high GC loading efficiency, high GC-to-lipid ratio, and unique slow controlled (zero order) GC release. The latter may improve the ability of the drug to reach non-phagocytic cells (e.g. T cells) [5] involved in the pro-inflammatory symptoms of CM [16].

Our goal was to design an efficacious protocol for the treatment of experimental cerebral malaria (ECM) which aimed to ameliorate or prevent the cerebral symptoms and create the therapeutic time-window necessary for administration of effective antiplasmodial drugs. In addition, our goal was to study the mechanism of action of GC, specifically BMS and nSSL-BMS, in the treatment of ECM, focusing on the brain inflammation. It is well established that in the context of neuroinflammatory disease, GC modulate T cells, macrophages, microglia, and the blood-brain barrier (BBB) [17]; these activities could all have potentially significant roles in the case of ECM. Parameters examined in our study include the biodistribution profile of GC administered as free drug or in nSSL. In parallel we analyzed gene expression in the inflamed brain of ECM mice as well as the development of cerebral edema, hemorrhages, and glial activation.

Our novel steroidal nano-drugs reduced (MPS-based) or eliminated (BMS-based) the development of ECM, creating the desired therapeutic time-window for the efficacious use of antiplasmodial drugs. This beneficial effect was achieved even under conditions in which treatment with free drug had no therapeutic effect.

## Materials and Methods

### Materials

#### Lipids

Hydrogenated soybean phosphatidylcholine (HSPC) was obtained from Lipoid KG (Ludwigshafen, Germany). Our HPLC analysis revealed that the HSPC is a mixture of mainly two PCs that constitute >98% of the total HSPC, with ∼28 mole% palmitoyl stearoyl PC (PSPC) and 61 mole% di-stearoyl PC (DSPC) [18]. It has a mid-temperature gel to liquid crystalline phase transition (T_m_) of 52.5°C. Cholesterol (>99% pure) was obtained from Sigma (St. Louis, MO, USA). *N*-(carbonyl-methoxypolyethyleneglycol-2000)-1,2-distearoyl-*sn*-glycero-3-phospho-ethanolamine sodium salt (2000-PEG-DSPE, referred to as PEG-DSPE) was obtained from Genzyme Pharmaceuticals, Liestal, Switzerland.

#### Drugs

MPS (Solu-medrol®) was obtained from Pfizer (Puurs, Belgium). BMS was custom synthesized for us by Steraloids (Newport, RI, USA). Hydrocortisone succinate sodium salt (HC), quinine, chloroquine, artemisinins, and dihydroartemisinin were obtained from Sigma. Artemisone was synthesized from dihydroartemisinin and purified by flash column chromatography, followed by recrystallization according to the procedure previously reported [19].

#### Water

Highly pure sterile and pyrogen-free water (18.2 MΩ, with less than 1 ppb total organic carbon) was obtained using the Clarkson D11951 Deionization System NANOpure(R) Diamond™ UV TOC Barnstead / Thermo Scientific (Midland, ON, Canada). DNAse-free ultra-pure water (UPW) was obtained from Biological Industries (Beit Haemek, Israel).

#### Other materials

HPLC-grade ethanol and acetonitrile were obtained from BioLab Ltd (Jerusalem, Israel). Glacial acetic acid was obtained from Frutarom (Haifa, Israel). Sorbitol, calcium acetate, sodium acetate, Dowex 2X-800 anion exchange resin (Cat. No. 60267-37-0) and normal goat serum (NGS) were obtained from Sigma. RPMI 1640, bicarbonate, hypoxanthine, gentamycin, and bovine serum albumin (BSA) were obtained from Biological Industries (Beit Haemek, Israel). Human plasma was obtained from the Hadassah Hospital blood bank.

### Animals

ICR Harlan-Sprague-Dawley (ICR) and C57Bl/6 Ola-Hsd male mice aged 7–8 weeks were obtained from Harlan Laboratories, Jerusalem, Israel.

### Ethical Treatment of Animals

All experiments were carried out in strict accordance with protocols approved by the Animal Ethical Care Committee of The Hebrew University of Jerusalem, Association for Assessment and Accreditation of Laboratory Animal Care (AAALAC) accreditation number #1285.

### Preparation of sterically stabilized nanoliposomes (nSSL) remote loaded with glucocorticosteroids (nSSL-GC)

nSSL remote loaded with MPS or BMS in 5% dextrose (w/v) with 10 mM histidine buffer (pH 6.8) were prepared according to our previously published protocol, using the described lipid composition (HSPC/cholesterol/PEG-DSPE at a mole ratio of 55∶40∶5) and transmembrane calcium acetate gradient (>1000) as the driving force for efficient and stable remote loading of the GC-succinate prodrugs [5]. The lipids were hydrated using 200 mM calcium acetate in the case of MPS loading and 250 mM calcium acetate in the case of BMS loading. The very low level of residual ethanol was determined using an ethanol assay kit (Megazyme ethanol determination kit, Megazyme International, Wicklow Ireland, Cat. No. K-EtOH; kit sensitivity limit 0.093 µg/ml). Calcium ion gradient determination was used as an in-process control before remote loading of drug. For this, intraliposome and extraliposome medium calcium concentrations were determined using flame atomic absorption spectrometry (FAAS; PERKIN-ELMER 403) or a calcium-ion selective electrode (Calcium Combination Electrode, Orion, Cat. No. 9720BNWP). The liposomes were sterile filtered and stored in sterile test tubes at 4°C until remote loading of drug. The chemical and physicochemical characterizations of the final product are described in Table 1.

**Table 1:** Characterization of nSSL before and after GC loading.

	After extrusion	After GC loading
**Size (z-average; d.nm)**	68.0±8.2	82.2±0.73
**Polydispersity index (PdI)**	0.14±0.02	0.12±0.01
**Residual ethanol, µg/ml**	not done	2.4±1.8
**CaAc gradient**	>1500	Remaining <250–500
**Osmolality, mOsm/kg**	7.00±0.04	6.80±0.03 (nSSL-MPS) 6.90±0.11 (nSSL-BMS)
**Zeta potential, mV**	(−3) – (−5)	(−3) – (−5)
**pH (formulation)**	4.5±0.5	4.4±0.8
**Final mM drug**	–	6.1±1.5 (nSSL-MPS) [3.2±0.8 mg/ml] 6.3±1.7 (nSSL-BMS) [3.0±0.8 mg/ml]
**Final drug:lipid ratio (mole/mole)**	–	0.21±0.08 (nSSL-MPS) 0.17±0.06 (nSSL-BMS)
**% drug encapsulated**	–	>95%

Values shown in each category are the average±SD for at least 10 formulations (13 empty nSSL, 27 nSSL-BMS, and 17 nSSL-MPS formulations). No significant differences were observed when comparing size according to intensity, number, or volume. No significant differences were observed when comparing nSSL-MPS and nSSL-BMS. PdI: an indication of size distribution variance between different batches prepared. A low PdI (<0.2) indicates that the sample is monodispersed.% drug encapsulated  = 100×([drug]/[lipid]_after Dowex anion exchanger_)/([drug]/[lipid]_after dialysis_).

The total and intraliposomal (after treatment with Dowex 2X-800 anion exchanger, pH 5, which binds all free drug) concentrations of MPS or BMS and their hydrolysis products were quantified using HPLC [5]. Liposome size distribution, phospholipid concentration, drug to lipid ratio, and level of free drug (i.e. percent drug encapsulated) were determined (see Table 1) as previously described. All liposomes used in mice were sterile and pyrogen free [5].

### P. falciparum culture

The parasite was grown in a human red blood cell culture (RPMI 1640, 7.5% bicarbonate, 50 µM hypoxanthine, 50 µg/ml gentamycin, and 7.5% human plasma) at 1% hematocrit and 2% parasitemia. Cultures containing predominantly ring forms were used; synchronization of the culture was performed if trophozoites represented more than 1–2% of the various developmental stages. Briefly, the culture was centrifuged and the supernatant replaced by 5% (isotonic) sorbitol. After 5 minutes at 37°C the culture was centrifuged again and the supernatant replaced by culture medium to obtain a final 1% hematocrit.

Drug testing was performed in 96-well microtiter plates incubated at 37°C in a 5% O_2_, 5% CO_2_, and 90% N_2_ gas mixture for 72 hours. After 48 hours, 1 µCi ^3^H-hypoxanthine was added to each well and the plates were read the next day using a scintillation counter.

### Induction of experimental cerebral malaria

*Plasmodium berghei* ANKA (PbA; PbA MRA-311, CDC, Atlanta, GA, USA) was maintained in vivo by serial transfer of parasitized erythrocytes (PE) from infected to naïve mice. Experiments were also performed using a second strain of PbA, similarly maintained, and designated PbAus in the text (courtesy of Prof. G. Grau, University of Sydney, Australia).

Intra-peritoneal injection of experimental mice with PbA or PbAus from peripheral blood of infected donor mice at an inoculum of 1−5×10^4^ PE resulted in 85–100% fatal ECM (depending on mouse strain) on days 9–11 post-inoculation (p.i.), at parasitemias of up to 20%. Parasitemia was monitored by thin blood smears prepared from tail blood. These were stained with a 25% Giemsa solution, examined under a light microscope, and parasitemia determined as the percent of infected red blood cells per 10,000 erythrocytes. Disease severity was determined according to scoring of neurological symptoms and changes in weight and temperature, as previously described [20] (Table 2). Parasitemias above 20% and the absence of neurological symptoms indicated severe anemic malaria.

**Table 2:** Scoring of clinical signs of ECM.

	Status	Score
**Appearance**	Normal	0
	Coat ruffled	1
	Coat staring; panting	2
	Dead	3
**Natural behavior**	Normal	0
	Hunched/wobbly gait	1
	Partial paralysis/immobile	2
	Convulsions/coma	3
	Dead	4
**Decrease in body weight**	0	0
	0–10%	1
	10%–15	2
	>15%	3
	Dead	4
**Body temperature**	36–37°C	0
	34–35°C	1
	32–33°C	2
	<32°C	3
	Dead	4

### Treatment protocols and mouse euthanasia

Mice were administered 5 mg/kg, 10 mg/kg, or 20 mg/kg free or nSSL-BMS by i.v. injection. For early ECM treatment, injections were given on days 3, 5, 7, and 9 p.i; for late treatment, treatment was given every other day, starting on day 5 p.i. for a total of at least two injections. Infected, non-treated control mice were injected with 5% dextrose or empty nSSL. Clinical signs and parasitemias were monitored and disease severity scored as described [20] (Table 1). Surviving mice were treated with 2×10 mg/kg/d artemisone by intraperitoneal injection in order to achieve cure; mice were monitored for four weeks post-artemisone treatment in order to allow detection of possible late recrudescence. Non-infected control mice were similarly injected with nSSL-BMS and sacrificed as above for immunohistochemical studies. At each time-point, at least 3 mice from each group were chosen at random, deeply anesthetized, and brains were removed following intracardial perfusion (using saline, if organs were destined for real-time (rt)PCR analysis, or saline/formalin, if organs were destined for histopathological analysis). Organs destined for rtPCR analysis were snap-frozen in liquid nitrogen and stored at −80°C until use. Organs destined for immunohistology were immediately immersed in cryoprotectant solution (see below).

### Isolation and preparation of total RNA

Relative gene expression was determined by rtPCR in the cortex and cerebellum, where most of the damage was noted in previous experiments (not shown). For total RNA extraction, samples were homogenized in Tri-reagent (Sigma) and the homogenate separated into aqueous and organic phases by chloroform addition and centrifugation. RNA was precipitated from the aqueous phase by addition of isopropanol, washed with ethanol, and solubilized in UPW. High RNA quality and purity was verified using a 1% agarose gel, and quantity determined using a spectrophotometer (ND-1000; Nanodrop).

### Polymerase chain reaction (PCR)

cDNA synthesis was performed using a High Capacity cDNA Reverse Transcription kit (Applied Biosystems, Warrington, UK). cDNA was amplified from 1 µg RNA from each sample with the addition of 2 µl Multiscribe™ reverse transcriptase (RT), 2 µl RNAse inhibitor, 4 µl random primers, 4 µl RT buffer, and 1.6 µl dNTP's according to the manufacturer's instructions for first-strand cDNA synthesis. UPW was added for a final sample volume of 20 µl. cDNA samples were stored at −20°C.

### Quantitative real-time PCR (qPCR)

Real-time transcription-polymerase chain reaction (rtPCR) was carried out using Applied Biosystems' 9800 Fast Thermal Cycler. The reaction mix for rtPCR analysis consisted of 1 µl cDNA, 300 nM forward (f) and reverse (r) primers (Syntezza, Jerusalem, Israel), and 7.5 µl manufactured master mix buffer containing nucleotides, Taq polymerase, and SYBR Green (Applied Biosystems) in a total volume of 15 µl. Gene amplification included one stage of 10 min at 95°C followed by 40 cycles of a two-step loop: 20 s at 95°C, and 1 min at 60°C. The fold-change in gene expression was normalized to the endogenous gene hypoxanthine phosphoribosyltransferase (HPRT). Relative quantification using the comparative (ΔΔCT) method was performed to values from non-infected mice; similar values were obtained in the cortex and cerebellum. Primers were designed using Primer Express (Applied Biosystems, Branchburg, NJ) and NCBI's primer-BLAST program, and their specificity and efficacy were validated (not shown). Results were considered significant if the observed fold-change was at least ×2 relative to non-infected mice. Primer sequences (5′-3′) were as follows: CCL5: TCACCATATGGCTCGGACACCACT (f), CACACTTGGCGGTTCCTTCGAGT (r); ICAM-1: GCCTCCGGACTTTCGATCTT (f); GTCAGGGGTGTCGAGCTTTG (r); IFN-γ: CAGCAACAGCAAGGCGAAA (f), GCTGGATTCCGGCAACAG (r); IL-4: CACAGGAGAAGGGACGCCATGC (f), TTGGAAGCCCTACAGACGAGCTCA (r); CXCL9: TTCCTTTTGGGCATCATCTT (f), ATCGTGCATTCCTTATCACT (r); CXCL10: GACGGTCCGCTGCAACTG (f), AGCTTCCCTATGGCCCTCA (r); HPRT: GCTTTCCCTGGTTAAGCAGTACA (f), CAAACTTGTCTGGAATTTCAAATC (r).

### Immunohistological studies

Organs destined for immunohistology were immediately immersed in cryoprotectant solution consisting of 30% sucrose in PBS for at least 48 h (cryoprotection was considered complete when the tissue no longer floated in the solution), after which they were encased in Tissue-Tek OCT compound (Bar-Naor, Petah Tikva, Israel) for storage at −80°C until transverse sectioning into ∼10 µ sections using a freezing microtome (cryostat, −20°C). Sections were thaw-mounted onto on poly-L-lysine-coated slides and stored at -80°C until use.

Slides were thawed by sequential incubation at −20°C and 5°C (10 minutes at each temperature) and incubated in blocking solution consisting of 20% of 5% BSA and 80% of 5% NGS, in a moist environment at room temperature (RT). After one hour the blocking solution was removed and sections incubated overnight at 5°C with primary antibody diluted 1∶200 in blocking solution. Microglia were stained using rabbit anti-mouse ionized calcium binding adaptor molecule 1 (IBA-1) (Wako Chemicals, Richmond, VA, USA); astrocytes were stained using rabbit anti-bovine glial fibrillary acidic protein (GFAP) (cross-reactive to mouse; LifeSpan Biosciences, Seattle, WA, USA). After washing in PBS, slides were incubated for one hour at RT with secondary antibody, Cy3-AffiniPure donkey anti-rabbit IgG H+L (diluted 1∶200 in PBS; Jackson Immunoresearch Laboratories, West Grove, PA, USA), followed by an additional wash. After addition of 4′,6-diamidino-2-phenylindole (DAPI) in mounting solution (Dapi-Fluoromount-G, Southern Biotech, Birmingham, AL, USA) the slides were mounted with coverslips and examined by fluorescent microscopy. At least three sections per brain (6 fields each) were examined, and the total number of astrocytes and microglia counted. High-resolution images of stained slides were photographed using a laser confocal scanning microscope. Images were visualized and analyzed with ImagePro analysis software: quantification of relative fluorescence was performed using the integrated optical density (IOD) parameter. IOD values of infected non-treated or treated mice were normalized to non-infected mouse values.

### Hematoxylin and eosin (H&E) staining

H&E staining was performed by incubation of slides in Sigma's Accustain hematoxylin solution followed by staining in eosin solution (2% eosin, 1% phloxine and 0.5% acetic acid in 95% ethanol), and mounting in Hydromount (National Diagnostics, Cherry Hill, NJ, USA).

### Evaluation of brain edema

BBB disruption was assessed by quantifying Evans blue dye extravasation [21]. 200 µl 2% (w/v) Evan's blue solution in PBS was injected i.v. to pre-warmed mice. After 2 h mice were anesthetized and perfused intracardially with ice-cold PBS. Brains were extracted, homogenized in 1 ml PBS, and centrifuged; the supernatant was read by spectrophotometry at 510 nm. Values were normalized to the values from the brains of non-infected mice similarly injected.

### Biodistribution of BMS administered as free BMS or nSSL-BMS

C57Bl/6 Ola-Hsd mice suffering from ECM were injected on day 6 p.i. with 20 mg/kg free or nSSL-BMS. Non-infected control mice were also injected with nSSL-BMS. At 1 h, 6 h, 24 h, and 48 h post-injection, mice (n = 3 per group) were anesthetized and sacrificed for organ collection. Organs were pooled and homogenized in 5% dextrose, followed by spiking with hydrocortisone, which served as an internal standard for extraction and sample processing. The homogenates were extracted as described elsewhere [22]. Quantification of BMS and its derivatives was performed by HPLC as described above.

### Statistics

When comparing parasitemia values or clinical scores, *p* values were calculated using Student's *t*-test or one-way ANOVA, respectively; for analysis of survival curves, the Kaplan-Meier test was employed. Values below 0.05 were considered significant.

## Results and Discussion

### Early administration of GC prevents ECM but does not prevent anemic malaria

**Figure 2:**
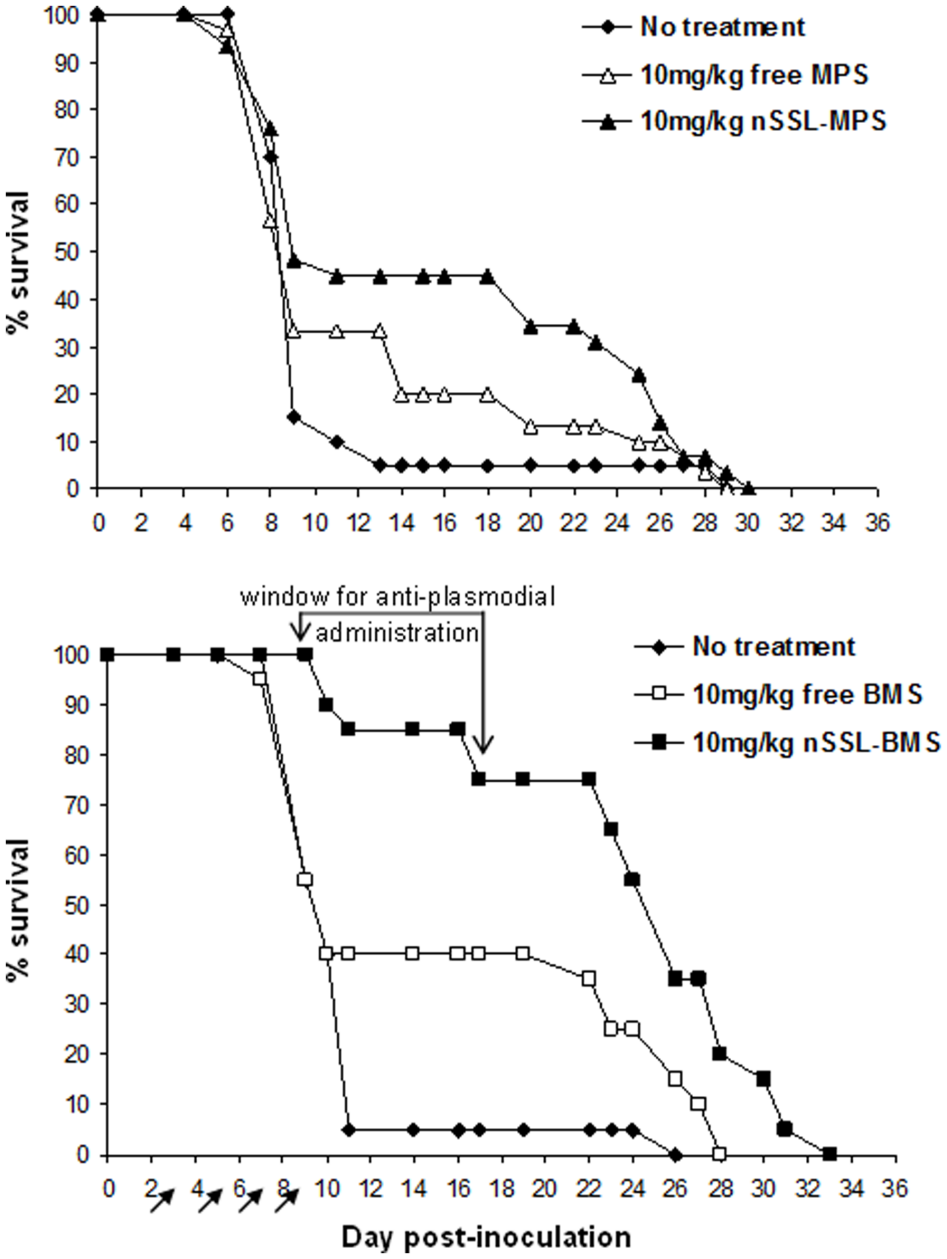
Survival rates after early treatment with 10 mg/kg free or nSSL-encapsulated MPS (upper graph) or BMS (lower graph). Representative results for ICR mice infected with PbA are presented. Arrows denote treatment administration. ECM prevention is reflected in longer survival times, due to the development of severe anemic malaria, and as a result creation of a survival time-window for anti-plasmodial administration. Significant differences in survival were seen between non-treated and nSSL-MPS-treated groups (p = 0.01) and between non-treated mice and mice administered free or nSSL-BMS (p<0.01).

Our general approach was to first establish whether GC administration can effectively eliminate/prevent ECM, and to determine which of the drugs, MPS or BMS, is superior for therapy. In our experiments, 95% of infected, non-treated C57Bl/6 mice died of ECM by day 11 p.i. Preliminary experiments showed that free MPS, at a dose of 25 mg/kg/d, displayed mild toxicity in ECM mice. For this reason, we chose a lower dose of 10 mg/kg/d for comparison of the effects of MPS and BMS. Treatment of infected mice with 10 mg/kg/d free MPS on days 3, 5, 7, and 9 p.i. had no significant effect on the rate of ECM development or survival of mice: 90% of mice were dead from ECM by day 11 p.i. (Figure 2). In contrast, administration of nSSL-MPS reduced ECM rates to 57%. On day 11 the survival rate of the nSSL-MPS-treated mice was 45% in comparison to 33% in the free MPS-treated group and 10% in the non-treated group (p<0.05). Mice saved from ECM by free MPS or nSSL-MPS treatment developed severe anemic malaria, as indicated by high parasitemia levels (above 20%). MPS was found to be weakly active against *P. falciparum* in vitro with an IC_50_ of 14 µM, significantly higher than the IC_50_ values of accepted antiplasmodial drugs (Table 3). No significant differences in the development of parasitemia were seen when comparing MPS-treated to non-treated mice (data not shown); thus, the in vivo effect of MPS is due to its immunomodulatory properties.

**Table 3:** Activity of designated antiplasmodial drugs and GC against cultured non-chloroquine resistant *P. falciparum*.

Drug	IC_50_
Quinine	0.38 µM
Chloroquine	0.04 µM
Artemisinin	0.13 nM
Dihydroartemisinin	0.10 nM
Artemisone	0.40 nM
MPS	14 µM
BMS	no effect*

* At concentrations up to 33 µM.

Administration of nSSL-BMS resulted in much better efficacy than that of nSSL-MPS. As in the previous experiment, the majority of control mice died of ECM by day 11 p.i. Treatment with 10 mg/kg/d free BMS decreased the incidence of CM from 95% to 67% (Figure 2). Administration of nSSL-BMS delayed the appearance of symptoms and further reduced ECM to only 15% (Figure 2). Mice that did not succumb to ECM died of severe anemic malaria three weeks later (Figure 2) and at high parasitemias (not shown). Free BMS was not effective against *P. falciparum* in vitro (Table 3), hinting at an immunological mechanism of action in vivo.

The greater therapeutic efficacy of BMS is in accordance with our previous experience in other disease models, in which the potency of BMS was five times higher than that of MPS [5]. Similar results following GC administration were obtained in both ICR and C57Bl/6 mice, using two different *P. berghei* strains (not shown). This indicates a robust treatment which is not dependent on specific mouse species and parasite strain combinations.

### The use of BMS encapsulated in nSSL drastically reduces drug toxicity and increases therapeutic efficacy

Our next step was to determine the most effective dose for early treatment (days 3, 5, 7, and 9 p.i.), using the more potent nano-drug, nSSL-BMS.

**Figure 3:**
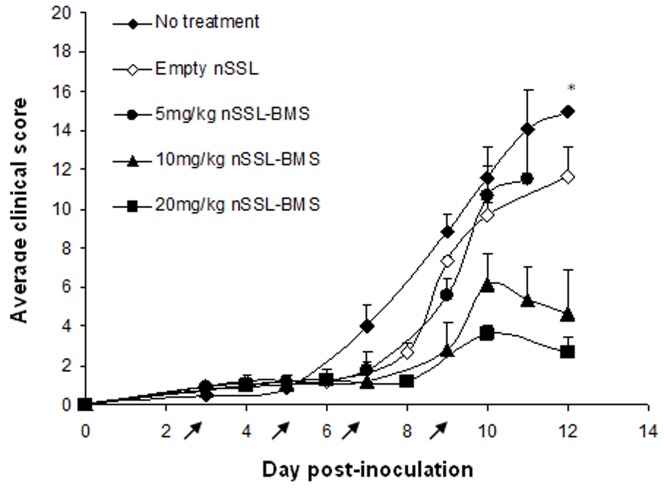
Effect of steroid treatment on clinical score during the ECM-susceptible phase of infection. PbAus-infected C57Bl/6 mice (n = 10, non-treated group, empty nSSL group, 5 mg/kg and 10 mg/kg nSSL-BMS groups; n = 13, 20 mg/kg nSSL-BMS group) were administered empty nSSL or nSSL-BMS on days 3, 5, 7, and 9 post-inoculation. A clear dose-response was seen, which translated to improved clinical score. Clinical scores of mice treated with 10 mg/kg or 20 mg/kg nSSL-BMS were significantly lower than those of control mice. *p = 0.8, non-treated vs. empty nSSL; p = 0.08, non-treated vs. 5 mg/kg nSSL-BMS; p<0.0001, non-treated vs. 10 mg/kg nSSL-BMS, non-treated vs. 20 mg/kg nSSL-BMS; p = 0.3, 10 mg/kg vs. 20 mg/kg nSSL-BMS. Arrows represent injections.

Treatment of ECM mice with free BMS at a dose of 5–20 mg/kg resulted in significant acute toxicity (Table 4). Mice that died after the first or second injection (days 3 or 5 p.i.) were not included in calculation of ECM incidence or clinical score. In cases of acute toxicity on days 7 or 9 p.i. the predicted fate of the animal (ECM or severe anemic malaria) was determined according to clinical signs on the day of death (i.e. low body temperature and significant weight reduction, 10% or more, indicated ECM). As in the previous experiments, no statistically significant effect on the prevalence of ECM was seen upon treatment with free BMS at doses up to 20 mg/kg/d; further increase in dose was not possible due to toxic effects of the treatment. No significant reduction in clinical score was seen, at any dose, following injection of free BMS (not shown). Injection of mice with empty liposomes had no effect on the development or progression of the cerebral syndrome: all mice succumbed to ECM by day 12 p.i., at clinical scores similar to those of non-treated infected control mice (Table 4, Figure 3). The use of nSSL-BMS completely abolished BMS toxicity and dramatically improved the efficacy of treatment: the incidence of ECM after administration of only 5 mg/kg/d nSSL-BMS was similar to that seen after treatment with 20 mg/kg/d free BMS (Table 4). Increased dose resulted in lower rates of ECM: administration of 10 mg/kg/d or 20 mg/kg/d nSSL-BMS resulted in ∼90% severe anemic malaria. Treatment with 10 mg/kg nSSL-BMS reduced the average clinical score (±SEM) from 14.5±1.5 in the non-treated group, 11 days post-inoculation, to 2.7±0.8 (Figure 3), compared to a reduction to only 7.5±1.6 in the free-BMS-treated group (not shown). Administration of 20 mg/kg BMS as nSSL-BMS similarly reduced disease severity to 5.4±1.7 (Figure 3), compared to 12.3±2.4 in the free BMS group (not shown). The difference in clinical score, when comparing 10 mg/kg and 20 mg/kg nSSL-BMS, was not significant (Figure 3).

**Table 4:** ECM rates after early treatment of PbAus-inoculated C57Bl/6 mice with free or nSSL-BMS.

		Death*				
Group	n	Toxicity	ECM		Severe anemic malaria	
5% dextrose	18	0	17	(95%)	1	(5%)
Empty nSSL	6	0	6	(100%)	0	(0%)
5 mg/kg free BMS	9	3	5	(83%)	1	(16%)
10 mg/kg free BMS	13	3	9	(90%)	1	(10%)
20 mg/kg free BMS	17	5	9	(75%)	3	(25%)
5 mg/kg nSSL-BMS	9	0	7	(78%)	3	(22%)
10 mg/kg nSSL-BMS	10	0	1	(10%)	9	(90%)
20 mg/kg nSSL-BMS	16	0	2	(12.5%)	14	(87.5%)

* The total number of mice per group excludes mice which died due to acute drug toxicity.

### Early administration of nSSL-BMS creates a time-window for antiplasmodial treatment and cure

The next step in our study was to validate the second part of our working hypothesis, namely that the extended survival time due to the elimination of ECM (Figure 2) can be used to treat the resulting anemic malaria with a conventional antiplasmodial drug. We selected an artemisinin-based drug, as artemisinin combination therapy is currently recommended by the World Health Organization as the most effective treatment for non-complicated malaria [23]. Artemisinin and its derivatives produce rapid clearance of parasitemia and rapid resolution of symptoms. However, problems with current artemisinin drugs include suspected toxicity [24] and evidence of emerging parasite resistance [25]. Artemisone is a new, second-generation semi-synthetic artemisinin derivative [19]. Artemisone is significantly more active than artesunate, the most widely available artemisinin derivative, both in vitro and in vivo, and has improved bioavailability and stability [26] with no known evidence of toxicity [19], [27].

**Figure 4:**
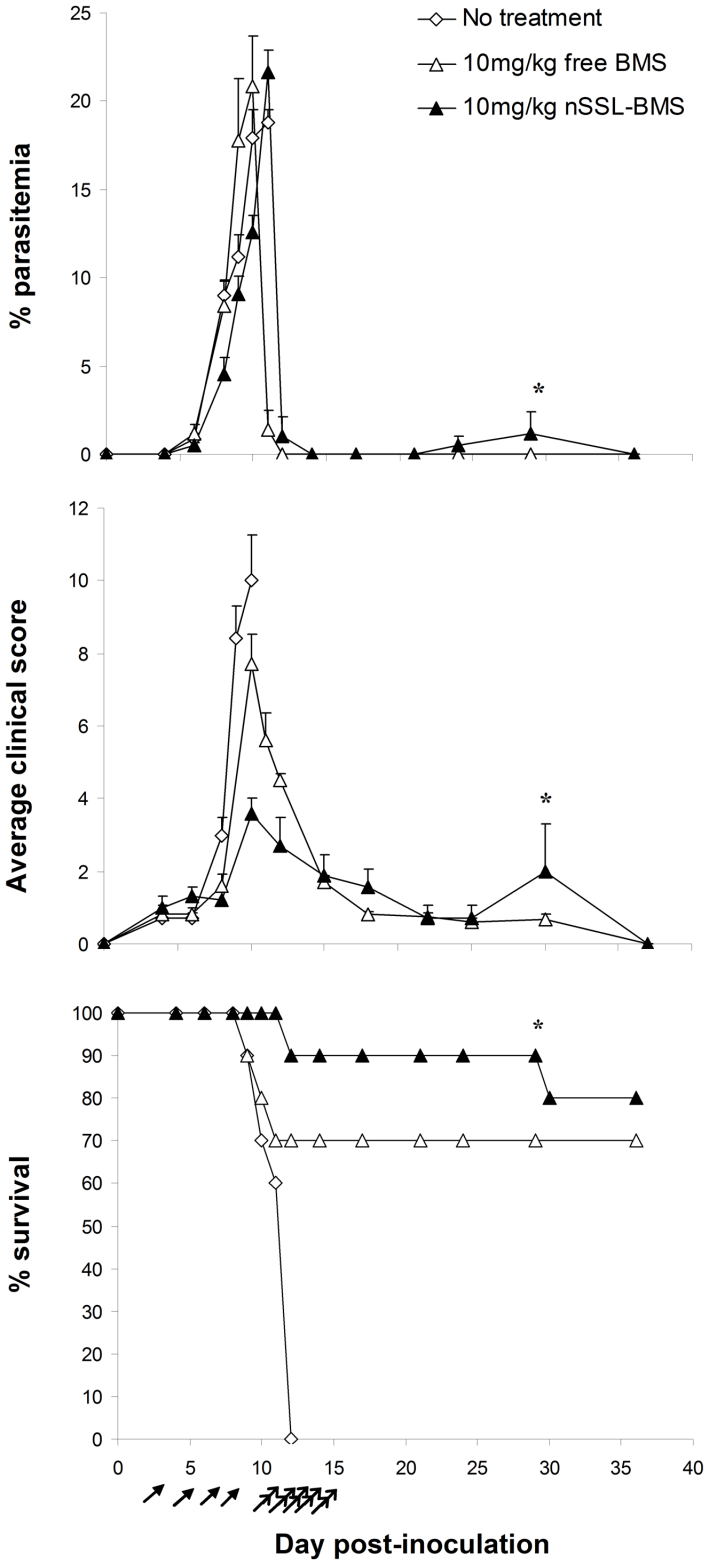
Effect of sequential steroid-artemisone treatment on the development of infection. PbAus-infected C57Bl/6 mice (n = 10 per group) were treated with 10 mg/kg free or nSSL-BMS on days 3, 5, 7, and 9 post-inoculation. Although parasitemias were not affected (p>0.05, all groups), treatment led to reduced clinical scores (p<0.05, non-treated vs. free BMS; p<0.001, vs. nSSL-BMS, days 1–12 p.i.), an effect more pronounced in liposome-treated mice (p<0.05, free vs. nSSL). Administration of 2×20 mg/kg/d artemisone on days 11–15p.i. led to cure. *One mouse (of the total 17 administered artemisone) relapsed and died of ECM. Arrows represent free or nSSL-BMS injections; double arrows represent artemisone injections.

Following early treatment with 10 mg/kg free or nSSL-BMS and the development of severe anemic malaria, as described above, we administered 2×20 mg/kg artemisone on days 11–15p.i., by i.p. injection. Artemisone treatment following nSSL-BMS administration resulted in a rapid drop to zero parasitemia, in the majority of cases within 24 h of the first injection, and led to complete cure: mice were monitored for recrudescence for four weeks after the end of treatment, and no secondary rise in parasitemia was observed (Figure 4). Namely, combination therapy leads to complete cure. Artemisone monotherapy beginning on day 11p.i. was ineffective: at this stage, all mice were at late stages of ECM and beyond the “point of no return”, at which it is impossible to rescue the mice by anti-plasmodial treatment (not shown).

### Administration of nSSL-BMS effectively prevents murine death from cerebral malaria even when administered at advanced disease stages

**Figure 5:**
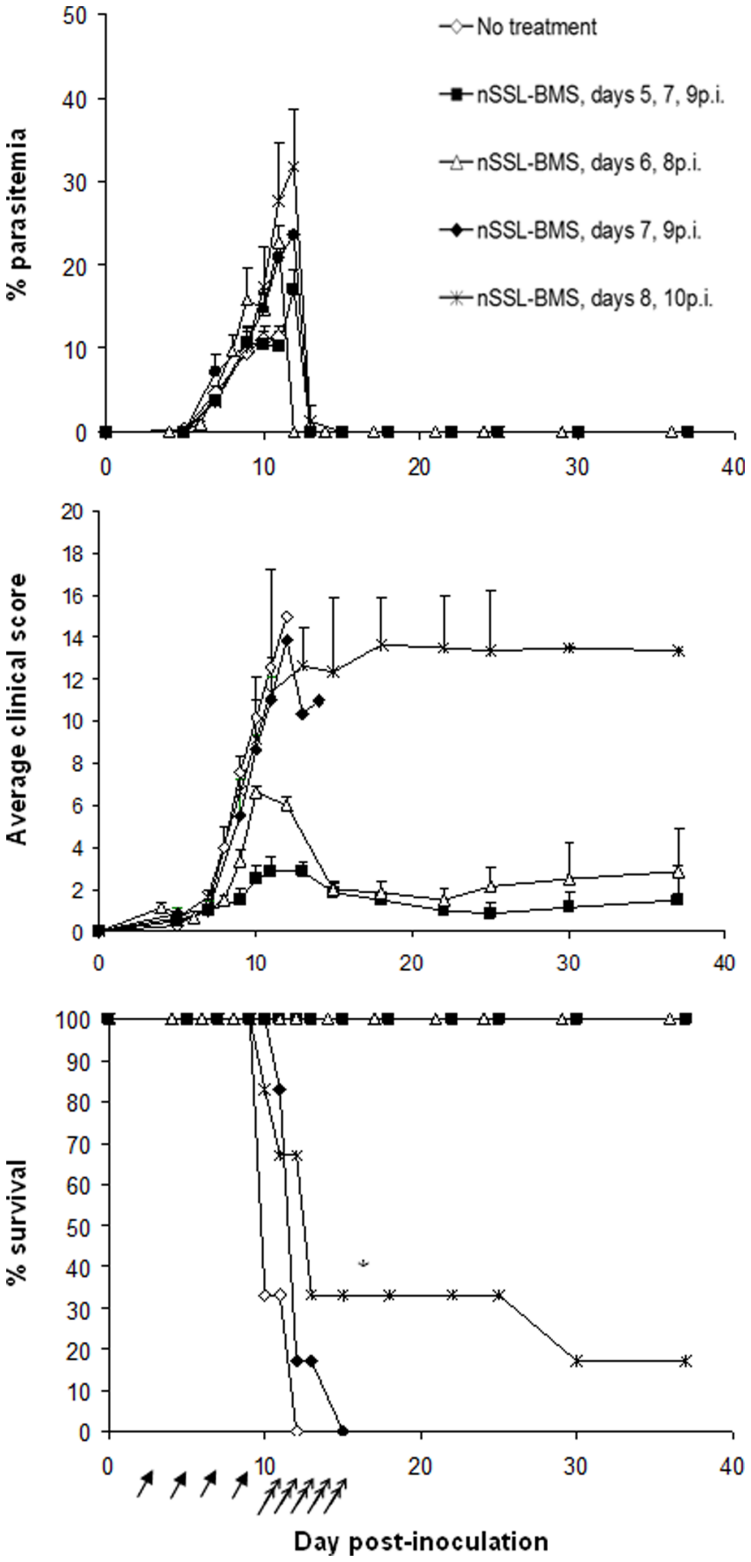
The effect of nSSL-BMS treatment at late stages of ECM, followed by artemisone. PbAus-infected C57Bl/6 mice (n = 10 per group, non-treated; n = 6 per group, nSSL-BMS) were administered 5% dextrose or 20 mg/kg nSSL-BMS (arrows) every other day, starting on day 5p.i., followed by 2×10 mg/kg/d artemisone after the cerebral phase, on days 11–15 p.i. (double arrows). No effect on the development of parasitemia was observed following administration of 5% dextrose. Administration of nSSL-BMS significantly reduced clinical score when started on day 5 p.i. or day 6 p.i. (p<0.001, p<0.05 vs. no treatment, respectively) and increased survival (p<0.01 and p<0.001 vs. non-treated), creating a time-window for antiplasmodial treatment and cure. *Day 15 p.i. nSSL-BMS: n = 6 (days 5,7,9 p.i. and days 6,8 p.i.).

Next we examined the efficacy of the BMS-artemisone combination therapy in the rescue of mice from late-stage ECM (Figure 5). We evaluated the therapeutic efficacy of nSSL-BMS administration at increasingly late stages of disease, after the appearance of blood parasitemia and clinical signs. C57Bl/6 mice (n = 6–10 per group) were inoculated with PbAus and monitored for parasitemias and disease progression. All non-treated control mice died of ECM on days 10–11 p.i., having an average parasitemia level of 11±1.7% and clinical score of 10±1.3.

Treatment was initiated in the first group on day 5p.i., when mice displayed low parasitemias but no clinical signs of ECM. Administration of 20 mg/kg free BMS on days 5, 7, and 9 p.i. did not prevent cerebral symptoms: on the contrary, 6/10 mice died of acute toxicity upon the second or third injection (days 7 or 9). The worsening condition of the mice in the free BMS group was exemplified by the average clinical score, which reached 4.2±2.2 on day 7 p.i., compared to 1.1±0.4 in the non-treated group on the same day. For this reason, we did not examine the effect of free BMS in any of the remaining treatment schedules. Administration of 20 mg/kg nSSL-BMS according to the same treatment schedule prevented ECM in all treated mice (n = 6): the average clinical score peaked on day 13 p.i. at only 2.8±0.4. Treatment with 2×20 mg/kg/d artemisone on days 11–15 p.i. led to complete cure: mice were monitored for three weeks after the end of treatment, and no recrudescence was detected.

Delaying the treatment to day 6 p.i (parasitemias 1.4±0.9%, clinical scores 1.5±0.7) did not decrease the efficacy of nSSL-BMS treatment: average scores the day after the end of treatment (day 9 p.i.) were 3.3±0.5, compared to 7.6±0.8 in non-treated mice. Subsequent administration of artemisone led to cure in all mice, with no recrudescence. We could not evaluate the therapeutic efficacy of free BMS administration due to its high toxicity (data not shown).

By day 7 p.i., parasitemias and clinical signs were moderate and rising (4.7±0.9% parasitemia; average clinical score 4±1). Treatment with nSSL-BMS, however, slightly delayed the rise in clinical score (average score on day 9 p.i. was 5.5±1.7, compared to 7.6±0.8 in non-treated mice) and prevented ECM in 5/6 mice. Administration of artemisone, however, was ineffective: all remaining mice died of severe anemic malaria.

Further delay of nSSL-BMS administration to day 8 p.i., when symptoms were severe (average parasitemia 10.2±2.5%, average score 10.1±1.9), resulted in cure in only 2 of the 6 treated mice; half of the mice in which ECM prevention was achieved died before completing artemisone treatment. We therefore consider day 7p.i. the “point of no return” after which the sequential administration of nSSL-BMS and artemisone is ineffective.

Overall, the results demonstrate that nSSL-GC administration effectively prevents ECM development and progression even at late disease stages, when severe clinical signs, such as ataxia and partial paralysis, are present. It is likely that had we commenced artemisone treatment earlier, or administered artemisone together with the nSSL-GC, complete cure would have been achieved in the majority of mice treated at these late stages.

We suggest that the combination of nSSL-BMS-artemisone therapy may have significant advantages compared to traditional antiplasmodial combination therapy. As described, the efficacious nSSL-BMS treatment averts ECM even at late stages of disease. A major drawback of most studies published regarding adjuvant therapy for ECM is the requirement for treatment at early disease stages [28]. In clinical practice, patients with CM are usually already in later stages of impaired consciousness or coma, and are treated immediately with antiplasmodial drugs, usually artemisinin combination therapies. In both ECM and CM the timing of treatment is highly significant: high efficacy at late stages of disease, when clear signs of cerebral damage are present, increase the chances for success. Our treatment regimen creates a much wider therapeutic window, enabling the use of the extended survival time gained to prevent the subsequent severe anemic malaria by administration of the antimalarial drug artemisone. These results might be relevant to human CM.

The suggested treatment regimen with artemisone is advantageous since, like other artemisinins derivatives, it is fast-acting (within 24 hours of the first injection) and although still in clinical trials, artemisone is the most therapeutically efficacious of these derivatives [20]. However, even with the recommended artemisinin-based therapy, mortality rates reach as high as 22% [29]. Clemmer et al. [28] report success rates of only 43–46% for ECM rescue therapy (after the appearance of clinical signs of neuropathology) using the artemisinin derivatives artemether or artesunate. Thus, adjunct therapies are gaining increased importance, especially for the preservation of neurological function during and after the treatment period. Treatment of CM using a drug with both antiplasmodial and anti-inflammatory properties has been suggested [30]. The potential benefit of adjunctive therapies in reducing neurological and cognitive dysfunction, common in CM survivors [1], has recently been demonstrated using murine models. Antioxidant therapy, as an additive to chloroquine, prevents the cognitive impairment in mice treated for ECM [31]. Adjuvant therapy of ECM using a synthetic host-defense peptide analogue together with antiplasmodial treatment was significantly more protective against late-stage infection than antimalarial treatment alone; this effect was linked to reduced inflammation [32]. Among their pleiotropic effects, steroids are recognized to have both anti-inflammatory and antioxidant activities [33]. Thus, the use of combination therapy which includes BMS may be especially appropriate for the prevention of neurocognitive impairment.

### Early treatment with BMS leads to reduced cerebral inflammation, edema, and hemorrhages

Following the establishment of an effective treatment regimen, we examined the specific effects of BMS and nSSL-BMS administration in the brains of mice bearing ECM. PbAus-inoculated C57Bl/6 mice were treated on days 3, 5, 7, and 9 p.i., with free or nSSL-BMS, followed by artemisone, as previously described. The infection resulted in significant increases in the expression of pro-inflammatory cytokines, chemokines, and ICAM-1 as was demonstrated on day 9 p.i. (Table 5), when clinical signs of ECM were at their peak. All non-treated disease-bearing mice died of ECM by day 11 p.i. Overall, although significant differences were observed in the incidence of ECM when comparing free- and nSSL-BMS-treated mice (compare 75% vs. 10% ECM, respectively), no significant differences in gene expression were noted when comparing the two groups, except in the case of CXCL10 (see below).

**Table 5:** Effect of treatment on expression levels of inflammation-related genes on day 9 p.i., as measured by qPCR.

	Relative expression ± SEM					
	CCL5	CXCL9	CXCL10	ICAM-1	IFNγ	IL-4
**No treatment**	129.0±8.4	39.4±32.1	21.4±6.3	15.2±4.1	192.8±21.3	1.2±0.6
**Free BMS**	45.7±7.6*	39.3±11.1	18.2±2.4	3.3±0.7*	105.1±37.7**	1.5±0.3
**nSSL-BMS**	46.3±23.6*	55.8±30.6	46.4±18.9*	5.9±2.6*	68.3±25.1**	2.4±1.4

PbAus-infected C57Bl/6 mice were administered free or nSSL-BMS on days 3, 5, 7, and 9 p.i. Administration of nSSL-BMS reduced the relative expression of CCL-5, ICAM-1, and IFNγ, indicating lower brain microvessel endothelial activation and cytokine levels. *p<0.01 vs. non-treated mice; **p<0.05 vs. non-treated mice. Except in the case of CXCL10 (p<0.01) no significant differences in gene expression were noted when comparing the free-BMS- and nSSL-BMS-treated groups.

Administration of free- or nSSL-BMS resulted in smaller increases in the levels of CCL5 on day 9 p.i., compared to non-treated infected mice. The development of ECM is critically dependent on CD8^+^ T cells, which sequester in the brain vasculature. CCL5, upregulated in ECM, is produced by CD8^+^ T cells and macrophages, as well as by endothelial cells which interact with parasite antigens [34], [35]. Together with macrophage inflammatory protein-1 (MIP-1, or CCL3), CCL5 interacts with leukocyte CCR5, and mediates trafficking of infiltrating lymphocytes especially CD8^+^ T cells [36]. Increased migration of CCR5^+^ leucocytes to the brains of mice with ECM has been described, while reduced levels of CCL5 have been described in the brains of mice not susceptible to ECM [36]. Significant upregulation and the association of CCL5 with CCR5 is also a main immune modulator of human CM immunopathology, particularly in the cerebellum and cerebrum [36]. CCL5-recruited CCR5^+^ leucocytes adhere to the cerebral microcapillaries, obstruct the microcirculation, and cause localized necrosis [37]. Down-regulation of CCL5 would therefore be expected to result in reduced chemotaxis of leukocytes, especially CD8^+^ T cells, to the brain microvasculature, and decreased immunopathology.

Higher levels of CXCL10 (p<0.01) were seen in nSSL-BMS-treated infected mice, relative to non-treated mice (Table 5). Administration of free BMS, however, had no effect. No significant changes in the levels of CXCL9 were seen when comparing treated and non-treated infected mice (not shown). Although CXCL10, together with CXCL9, is induced by IFNγ and is required for the development of ECM, studies showing upregulation of these chemokines in non-cerebral (i.e. severe anemic) malaria indicate that they alone are insufficient to drive effector cell recruitment [38]–[40]. Our results are in accordance with these studies, as nSSL-BMS-treated mice were protected from cerebral pathology and displayed high levels of CXCL10 (Table 6), while levels in the free BMS group were similar to those of non-treated mice.

**Table 6:** Effect of treatment on brain pathology.

Day post parasite inoculation	Group	% survival	Group score	Edema, ng Evan's blue/brain	Number of hemorrhages	Microglia, normalized IOD*	Astrocytes, normalized IOD*
**5**	No treatment	100	0.8±0.8	149±59	203±4	15±1.4	2.6±0.2^†^
	Free BMS	100	1.3±1.1	263±95	9±1	12±1	1.7±0.9
	nSSL-BMS	100	1.0±0.1	0	181±12^††^	2.2±0.1^‡^	1.8±0.1
**9**	No treatment	45	8.6±1.6	1073±255^†^	0	86.7±1.6^†^	10.9±0.4^†^
	Free BMS	47	8.4 ±1.7	263±6	9±2	10±0.05	1.1±0.1
	nSSL-BMS	100	2.8 ±1.3	163±76	20±9	11.2±1.2	2.9±0.01^‡‡^
20**	nSSL-BMS	88	1.2±0.5	139±29	2±2	13.3±0.01	3.4±0.08

PbAus-infected C57Bl/6 mice were administered placebo (5% dextrose), free BMS or nSSL-BMS on days 3, 5, 7, and 9 p.i. Surviving mice were subsequently treated with 2×20 mg/kg/d artemisone on days 16–19 p.i., when parasitemia levels clearly reflected severe anemic malaria. Survival rates on day 13p.i., at the end of the cerebral phase, were 0 in the non-treated group and 11% in the free BMS group, vs. 88% in the nSSL-BMS group. **Survival in the non-treated and free-BMS-treated groups was 0 on day 20 p.i. *Integrated optical density. IOD values of IBA-1 (microglia) or GFAP (astrocytes) in infected non-treated or treated mice were normalized to non-infected mouse values. ^†^p<0.03, non-treated vs. treated mice; ^††^p<0.001, non-treated vs. treated mice. ^‡^p<0.01, vs. non-treated and free-BMS-treated mice. ^‡‡^p = 0.2, free vs. nSSL-BMS.

A dramatic rise in IFNγ and increased levels of ICAM-1 were seen after PbAus inoculation. Treatment with free or nSSL-BMS caused a significant reduction of this increase, in both IFNγ (p<0.05) and ICAM-1 (p<0.01) (Table 6). Although IL4 levels on day 9 p.i. in nSSL-BMS treated mice were double those in non-treated mice the fold-change was low, indicating little relevance (Table 5); administration of free BMS did not affect IL4 expression. Studies with brain microvascular endothelial cells have shown that the expression of ICAM-1 is proportional to the levels of IFNγ and inversely proportional to the level of IL4 [41].

IFNγ has been shown to be crucial to the development of ECM. CD8^+^ T cells and IFNγ drive the rapid increase in total parasite biomass and accumulation of iRBC in the brain 6–12 days post-infection, when mice develop ECM [42]. High levels of circulating IFNγ and upregulation of IFN-responsive genes are also correlated with development of CM in humans [3].

ICAM-1, expressed in the BBB and also synthesized by astrocytes, is also a critical mediator of cerebral pathology. Upregulation of endothelial receptors in the brain is the result of significant systemic increases in the levels of circulating pro-inflammatory cytokines [43], as observed in cerebral malaria [44]. In human CM the Th1 response leads to overproduction of IFNγ, which in turn upregulates receptors and stimulates TNF production by monocytes, leading to upregulation of ICAM-1 on brain endothelial cells. High levels of ICAM-1 cause increased adhesiveness of platelets and iRBC to brain endothelial cells and transmigration of leukocytes, especially T cells [45]. TNF-induced ICAM-1 expression in human brain endothelial cells was shown to be reduced by high doses of methylprednisolone [46] and dexamethasone [47].

iRBC sequestration in brain endothelium (without extravasation to the brain) is considered central to CM pathology and, concomitant with other brain-endothelium-derived cytokines/chemokines, likely contributes to the neurological pathology resulting in coma and seizures, with death in more than 20% of cases [48]. In vitro experiments have demonstrated that iRBC-mediated activation of the host BBB/endothelial cells leads to increased expression of luminal ICAM-1 and polarized release of cytokines/chemokine to both luminal and basal sides of the BBB [49]. Downstream effects include vessel obstruction, ischemia, and changes in normal metabolism, leading to neuronal dysfunction, vessel disruption leading to brain hemorrhages, and BBB disruption [50]. Mice deficient in ICAM-1 are protected from ECM, protection being associated with a decrease in adherent monocytes and iRBC, as well as an absence of BBB disruption [51]. In our experiments, the reduced IFNγ and ICAM-1 levels seen in BMS-treated mice were concomitant with significantly lower levels of edema and hemorrhages, compared to non-treated mice (Table 6; see below).

Edema was apparent in non-treated and free BMS-treated mice as early as day 5 p.i; no edema was seen at this stage in the brains of mice administered nSSL-BMS (Table 6). Edema levels in mice treated with free BMS were similar to those seen in non-treated mice. By day 9 p.i. significant levels of edema were seen in non-treated mice, correlating with advanced stages of ECM: the average clinical score was 8.6±1.6, and the mice were immobile, displaying drastically reduced body temperatures and partial paralysis. In nSSL-BMS-treated mice, low levels of edema appeared on day 9 p.i., when clinical signs were mild. Edema levels remained unchanged in nSSL-BMS treated mice cured as a result of artemisone administration (on days 11-15 p.i.). Clinical scores on day 20 p.i. were low (1±0.4), indicating that the residual edema did not cause acute neurological damage.

**Figure 6:**
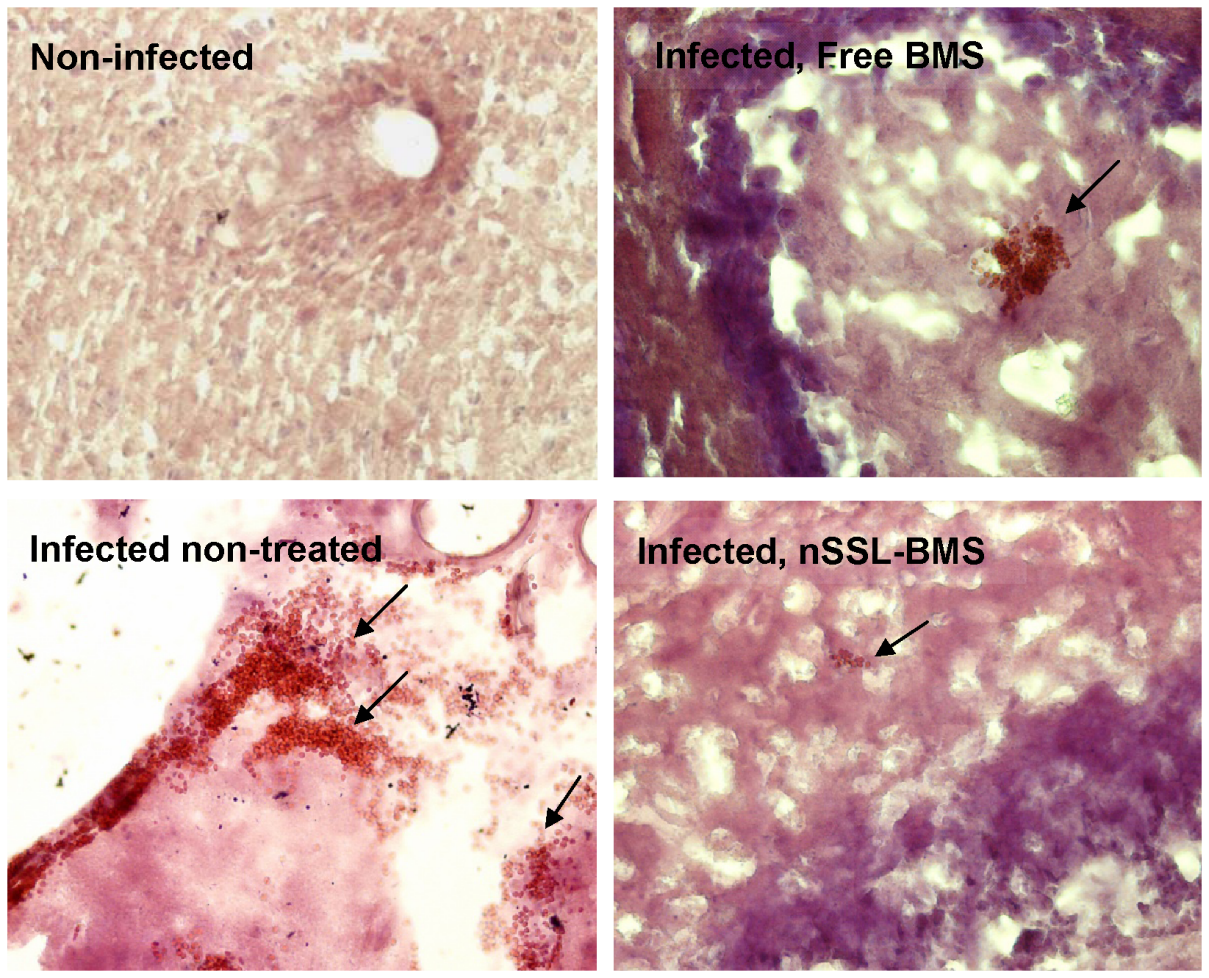
Hemorrhages post-PbAus infection and following free BMS or nSSL-BMS administration. H&E staining: representative pictures depicting hemorrhage size, day 9 p.i. (magnification ×20). No hemorrhages were observed in the brains of healthy mice. PbAus-infected C57Bl/6 mice were administered placebo (5% dextrose, n = 22), 20 mg/kg free BMS (n = 17) or nSSL-BMS (n = 18) on days 3, 5, 7, and 9 p.i. Survival rates on day 13p.i., at the end of the cerebral phase, were 0 in the placebo group and 12% in the free BMS group, vs. 88% in the nSSL-BMS group.

Significant, widespread hemorrhage was seen in the brains of infected non-treated mice (Table 6, Figure 6). In these mice, hemorrhage on day 5 p.i. was non-uniform, and both small (10–25 RBC) and major hemorrhages were observed; by day 9 p.i. hemorrhaging was severe and widespread. BMS-treated mice displayed significantly fewer and less severe hemorrhages. On day 5 p.i. the degree of hemorrhaging in treated mice was negligible (few cases, of <5 RBC); on day 9 p.i., mostly small hemorrhages (10–20 RBC or less) were seen, and by day 20 p.i. almost no hemorrhages were seen although capillaries remained packed and slightly enlarged.

In light of these results, we hypothesize that in mice suffering from ECM, the cerebral inflammation together with the mechanical damage to the BBB led to edema, raised intracranial pressure, hemorrhages, and death. This is in accordance with previous results demonstrating the role of CD8^+^ T cells in perforin-mediated apoptosis of cerebral microvascular endothelial cells [52], and recent research demonstrating cerebral vascular collapse in ECM [53]. Dexamethasone has been shown to bind membrane-linked GC receptors on T cells, leading to inhibition of TCR signaling, [54] with the downstream effect of reduced perforin activity and perforin-mediated damage. Indeed, nSSL-BMS-treated mice displayed reduced inflammation, edema, and hemorrhaging.

### Administration of nSSL-BMS leads to reduced glial cell death

The BBB regulates the passage of essential components and microorganisms between the bloodstream and the brain parenchyma. The BBB also controls the uptake of nano-drugs: in mice with inflammation-induced compromised BBB there is increased nSSL uptake [55]. The anatomical constituents of the BBB are the endothelial cells, pericytes, and basal lamina (matrix proteins) that together with astrocytes, neurons, and microglia, comprise the neurovascular unit [56]. Immune privilege of the CNS is maintained by the tight endothelial junctions of the BBB, the absence of adequate connections with the immune system, and the presence of an immunosuppressive microenvironment. This strict regulation of CNS immune reactivity is compromised in neuroinflammatory disorders, in which large numbers of leukocytes are recruited to the CNS, often leading to irreversible neurological impairment [57].

Of hematopoietic origin, microglia play a major role in homeostasis and repair: they possess phagocytic and antigen presenting properties and are involved with both innate and adaptive immunity, regulating inflammation and cell damage via activation of Toll-like receptors and cytokine production [58]. Microglial activation can result in the production of either pro- or anti-inflammatory cytokines [57] as well as nitric oxide [59], leading to neuroinflammation and NO-mediated neurotoxicity. In CM, however, the role of NO is debatable; the common view is that high levels of NO are associated with a protective effect [60].

Among the earliest changes observed in the brain parenchyma following PbA inoculation are changes in microglia. Approximately three days before the onset of cerebral symptoms, morphological changes including ramified processes, enlargement, an increasingly amoeboid appearance, and vacuolation are seen. Redistribution of activated microglia towards retinal vessels is also observed [61]. The initiating event for microglial activation appears to be the increased BBB permeability and the resulting exposure to plasma constituents, including pro-inflammatory cytokines such as IFNγ and TNF.

**Figure 7:**
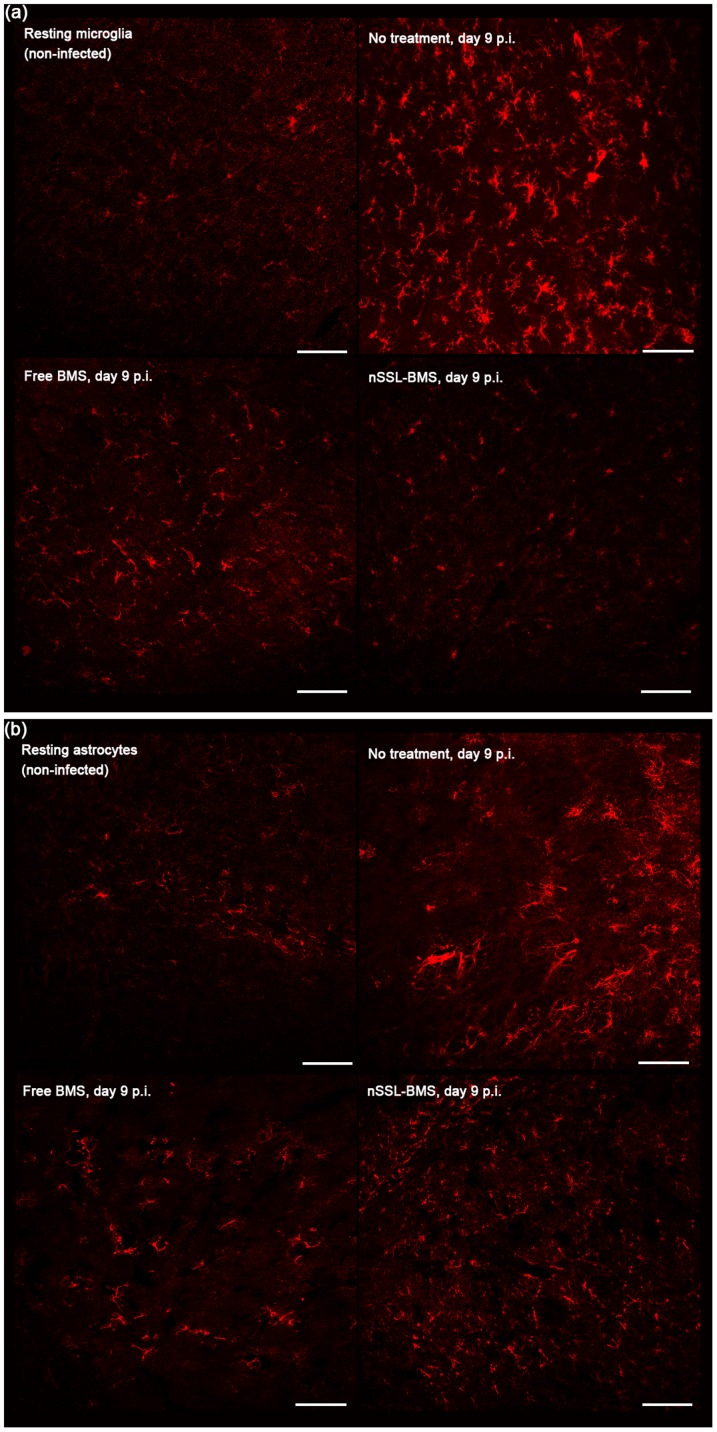
Reduction in (a) microglial and (b) astrocyte activation in PbAus-infected C57Bl/6 mice treated with nSSL-BMS. Infected mice were administered placebo (5% dextrose, n = 22), 20 mg/kg free BMS (n = 17), or nSSL-BMS (n = 18) on days 3, 5, 7, and 9 p.i. Infection with PbAus induced significant activation of microglia (7a) and astrocytes (7b). Administration of nSSL-BMS resulted in reduced microglial and astrocyte activation and cell numbers; a lesser effect was observed after treatment with free BMS. 3–5 mice were examined at each time-point. Scale bar 100 µm.

In our experiments, PbAus infection led to significant increases in microglial activation as early as day 5 p.i. (Table 6); most of the microglia were localized in the outer layers of the brain, closer to the brain surface. Significant migration and redistribution of microglia was observed in non-treated mice: by day 9 p.i. the activated cells were uniformly distributed throughout the brain. In free-BMS-treated mice. a lower level of activated cells was seen on day 5 p.i.; by day 9 p.i. significant cell enlargement was observed (not shown), concomitant with a vast increase in their number. In nSSL-BMS-treated mice, microglia were also activated on day 5 p.i., as demonstrated by numerous elongated processes, but they remained small in size and uniformly distributed throughout the brain. On day 9 p.i. microglia in non-treated mice had enlarged cell bodies as well as shorter and thicker processes; average cell body size was 50 µm, vs. 10 µm in nSSL-BMS-treated mice, the latter of which resembled resting microglia in non-infected mice (Figure 7a). The high level of microglial activation seen in the infected non-treated group is in accord with other results indicating disruption of the BBB (Table 6). The clinical score for the nSSL-BMS treated group on day 9 p.i. averaged 2.8±1.3, vs. similar high scores of 8.4±1.7 in the free BMS group and 8.6±1.6 in the non-treated group, another indication of the therapeutic efficacy of nSSL-BMS administration (Table 6). Our results are in agreement with previous publications reporting a correlation between reduced microglial activation and reduced cerebral symptoms [62].

Astrocytes, ubiquitous throughout brain tissue, make essential contributions to many homeostatic functions that directly influence neuronal survival and tissue integrity and are key cells involved in mediating the inflammatory process in the brain [63]. Due to their wide range of activities, both harmful and beneficial effects have been attributed to activated astrocytes. The induction of chemokines and adhesion molecules in astrocytes is likely to make a major contribution to the recruitment and retention of leukocytes in the CNS, the prologue to neuropathology [63].

Low numbers of astrocytes, mainly in the cerebral ventricles and outer cortex, were seen in non-infected mice. PbAus infection caused an increase in astrocyte activation (Figure 7b) and significant migration toward the ventricles. It has been suggested that changes in astrocyte distribution are the result of increase in BBB permeability [64]. Edema was indeed evident in non-treated ECM mice by day 5 p.i. (Table 6). On day 9 p.i., significantly higher numbers of astrocytes were seen in non-treated mice; cell activation was evidenced by the intensity of staining (Figure 7b) as well as cell morphology: reactive astrocytes had an increased number of primary processes (77±4 processes in infected non-treated mice on day 9 p.i. vs. 18±4 on day 5p.i and 8±3 in non-infected mice; n = 41, 42, and 42 cells, respectively). Hypertrophy of intermediate filament-rich main cellular processes is indicative of astrocyte activation [65]. Lower numbers of astrocytes were seen in both free- and nSSL-BMS-treated mice on day 9p.i., relative to non-treated mice, and the degree of reactivity appeared to be reduced, as evidenced by reduced process hypertrophy (10±2, 12±3; n = 23, 18 cells, free or nSSL-BMS, respectively) and finer processes. Astrocytes were localized primarily to cerebral microvessels. On day 20 p.i., in mice cured with artemisone subsequent to nSSL-BMS administration, astrocytes were localized primarily to the cortex and the number of processes around blood vessels remained high.

Astrocyte activation has been correlated with severe edema in ECM development [66]. The most abundant aquaporin in the brain, aquaporin 4 (AQP4) is a transmembranal protein which acts as a bidirectional water channel. Upregulated in the foot processes of astrocytes at BBB- and cerebrospinal fluid-brain interfaces, AQP4 facilitates water movement into astrocytes in cytotoxic edema, and water movement out of the brain in vasogenic edema; both types of edema occur in ECM. Although astrocyte AQP4 is upregulated in both ECM and severe anemic malaria, expression levels are significantly higher in ECM, where AQP4 appears to have a pathogenic role [66]. Our results demonstrating high levels of astrocyte activation and significant edema in non-treated mice with cerebral symptoms, compared to reduced astrocyte activation and lower edema in treated mice, are in line with the proposed contribution of astrocytes to the development of edema in ECM.

### The high level of stable encapsulation of BMS in nSSL enables its selective accumulation in the brains of diseased mice

Table 7 summarizes the differences in time-dependent distribution of BMS injected i.v. as nSSL-BMS to normal healthy mice and as free BMS or nSSL-BMS to ECM-bearing mice on day 6 post-infection. In the infected groups, parasitemias were ∼1%, the mice had lost 9% of their initial weight, and the average body temperature was 35.8°C, indicating initial stages of ECM.

**Table 7:** Levels of BMS originating from nSSL-BMS or free BMS in tissues of naive healthy mice and mice with ECM.

		BMS, µg drug/g organ		
		Non-infected mice	Infected mice	
Organ	Time post-injection, h	nSSL-BMS	nSSL-BMS	Free BMS
**Brain**	1	0	0.2	0.4
	6	0	0.4	0.1
	24	0	0.6	0.1
	48	n.d.*	1.4	0.1
**Liver**	1	0.1	1.7	2.4
	6	0.4	2.5	3.4
	24	0.2	0.7	0.4
	48	n.d.	1.3	0.4
**Lungs**	1	1.6	1.9	1.6
	6	2.2	2.1	2.4
	24	2.4	1.6	1.9
	48	n.d.	5.7	2
**Heart**	1	3.2	4.1	2.9
	6	6	3.1	2.4
	24	0.4	2.4	3
	48	n.d.	8.0	3.3

Mice at initial clinical stages of ECM were injected (on day 6 p.i.) with either free or nSSL-BMS. Healthy mice were injected with nSSL-BMS. *n.d, not done.

The disease led to significant increases in the levels of drug in the brain at every examined time-point. Overall, during the 48 hours post-drug injection the diseased brain was exposed to much greater drug levels than the brains of healthy non-infected mice, in which the BMS level was below the detection limit (0.01 µg BMS). This can be well correlated with the high potential for therapeutic efficacy selective for the infected brain while not reaching, and therefore being non-toxic to the brains of non-infected mice. This supports the suggestion that the damaged BBB in the diseased mice is the “Achilles Heel” of the disease which can be utilized to treat the cerebral pathology. A similar effect was previously demonstrated in the case of experimental autoimmune encephalomyelitis [5]. No less important are the significant differences in the profiles of BMS accumulation in the brain when comparing the free- and nSSL-BMS-treated groups. In diseased mice injected with free BMS a short-lasting increased accumulation of BMS in the brain was noted at 1 hour post-injection (0.4 µg/g wet tissue) and levels returned to background at some time-point between 1 and 6 hours. In the nSSL-BMS group the concentration increased at a slower rate, but steadily, and BMS continued to accumulate for the next 48 hours, reaching a peak level of 1.4 µg/g wet tissue at 48 h post-injection. These large differences between free and liposomal GC are explained by the large reduction of drug clearance and higher plasma concentrations of the liposomal drug when compared with the free drug [5]. Even more important is the finding that at 6 h post-injection, the brain to liver BMS concentration ratio was ∼5.5 times higher for nSSL-BMS, compared to free drug (0.16 vs. 0.03); by 24 h post-injection the ratio in free-BMS-treated mice had risen 1.5-fold, compared to 7.3-fold in the nSSL-BMS group. As in the comparison of nSSL-BMS in infected and non-infected mice (Table 7), our results depict selective accumulation of BMS only in the brains of diseased mice, which is consistent with the BBB damage known to occur in ECM. The higher drug levels in the brain upon injection of the liposomal nano-drug, when compared with free drug, demonstrate passive drug targeting to injured areas in the brain.

Apart from the brain, significant differences in the amount of drug accumulation, over time, were seen when comparing the livers of disease-bearing and healthy mice (peak concentrations at 6 h post-nSSL-BMS injection of 2.5 µg/g vs. 0.4 µg/g BMS, respectively) (Table 7). This 6-fold higher concentration in disease-bearing mice may be partially due to the damage caused to the liver, one of the sequestration sites of both iRBC and leukocytes during malaria [67]. In ECM mice, the level of BMS in the liver was 1.4-fold higher, one hour and six hours post drug injection, in mice treated with free BMS, when compared to mice injected with nSSL-BMS (in which the drug is liposomal, and therefore less available). At the same time very little BMS was detected in the liver of the naïve healthy mice. This difference may be partially due to the damage caused to the liver by the disease [67]. This may also indicate lower rates of first-pass metabolism of the liposomal BMS, which may be expressed as lower GC side effects [68]. Since the treatment with free BMS had toxic effects and was far less effective than nSSL-BMS administration, the level of the BMS in the liver may indicate the degree of its toxicity, as similar quantities of BMS were injected to both groups.

Differences in the accumulation of BMS in the lungs and heart were also noted (Table 7). In the hearts of healthy mice, BMS levels peaked at 6 µg/g six hours post-injection. In contrast, accumulation of BMS in ECM mice was more gradual, reaching a plateau of ∼3 µg/g 24 hours post-injection in mice administered free BMS, and 8 µg/g 48 hours post-injection in mice administered nSSL-BMS. In the lungs of ECM-bearing mice, BMS levels reached a plateau of 2 µg/g in the free BMS group vs. 5.7 µg/g in the nSSL-BMS group, 24 and 48 hours post-injection, respectively. The differences in drug accumulation, when comparing healthy and sick mice, may be explained by damage to these organs caused by the disease. Human CM may be accompanied by acute lung injury and acute respiratory distress syndrome, and although the focus is usually on neurological complications, PbA infection in mice causes multi-organ disease, particularly in the lung [69]. Hydrothorax/pleural effusion may also occur in both human and murine malaria in addition to cerebral symptoms, indicating damage to the heart [70]. The higher levels of BMS upon administration of nSSL-BMS, 48 h post-injection, may be due to the inherent advantage of the nSSL in delivery of the drug to the organs affected by the disease.

## Conclusions

### Treatment of ECM: Comparing GCs and nSSL-GCs

1 nSSL-BMS is an efficacious modality for the treatment of ECM, and when followed by the antimalarial drug artemisone it enables a complete cure of the malarial symptoms.
2 MPS and BMS were selected as the GCs of choice due to their high anti-inflammatory efficacy and the fact that both are amphipathic weak acids, which allowed us to achieve highly efficient remote loading into nSSL. BMS was preferred over MPS because it showed higher potency for treatment of ECM in its free (non-liposomal) form. However, the use of BMS as free drug is problematic due to its much higher toxicity and resulting poor therapeutic index. Encapsulation of BMS in nSSL to form the nano-steroidal drug nSSL-BMS prevents toxicity and therefore improves BMS therapeutic efficacy, making nSSL-BMS the nano-drug of choice.
3 nSSL-BMS reduces ECM rates in a dose-dependent manner.
4 Administration of nSSL-BMS before the appearance of clinical signs prevents the development of ECM and results in improved clinical scores and increased survival time.
5 Administration of nSSL-BMS after the appearance of clinical signs effectively prevents ECM even when treatment is started at late disease stages, when damage to the blood-brain barrier has already occurred.
6 Administration of nSSL-BMS or nSSL-MPS does not affect parasitemia levels, and treated mice develop severe anemic malaria.
7 nSSL-BMS (but not nSSL-MPS) treatment enables a significantly wider survival time-window for antiplasmodial treatment (such as artemisone). Consequently, treatment of severe anemic malaria with artemisone, post nSSL-BMS administration, leads to complete disease cure.

The differences in the MoA of free and nSSL-BMS are explained by the following findings:

8 Treatment with nSSL-BMS, but not free BMS, effectively delivers and releases BMS in the brain of ECM-diseased mice.
9 BMS affects inflammatory cytokines, chemokines, and adhesion molecules, and effectively ameliorates cerebral inflammation, hemorrhage, and edema, in accord with reduced microglia and astrocyte activation.
10 The superiority of the nSSL-BMS nano-drug, given in combination with the potent antimalarial drug artemisone, is a highly efficacious treatment for ECM, making it attractive for investigation for the treatment of human cerebral malaria.
